# Recombinant *Ralstonia eutropha* engineered to utilize xylose and its use for the production of poly(3-hydroxybutyrate) from sunflower stalk hydrolysate solution

**DOI:** 10.1186/s12934-016-0495-6

**Published:** 2016-06-03

**Authors:** Hee Su Kim, Young Hoon Oh, Young-Ah Jang, Kyoung Hee Kang, Yokimiko David, Ju Hyun Yu, Bong Keun Song, Jong-il Choi, Yong Keun Chang, Jeong Chan Joo, Si Jae Park

**Affiliations:** Department of Chemical and Biomolecular Engineering (BK21 Plus Program), KAIST, 335 Gwahagno, Yuseong-gu, Daejeon, 34141 Republic of Korea; Center for Bio-based Chemistry, Division of Convergence Chemistry, Korea Research Institute of Chemical Technology, P.O. Box 107, 141 Gajeong-ro, Yuseong-gu, Daejeon, 34602 Republic of Korea; Department of Environmental Engineering and Energy, Myongji University, 116 Myongji-ro, Cheoin-gu, Yongin, Gyeonggido 17058 Republic of Korea; Department of Biotechnology and Bioengineering, Chonnam National University, 77 Yongbong-ro, Buk-gu, Gwangju, 61186 Republic of Korea

**Keywords:** Lignocelluloses, Sunflower stalk, Xylose, Poly(3-hydroxybutyrate), *Ralstonia eutropha*

## Abstract

**Background:**

Lignocellulosic raw materials have extensively been examined for the production of bio-based fuels, chemicals, and polymers using microbial platforms. Since xylose is one of the major components of the hydrolyzed lignocelluloses, it is being considered a promising substrate in lignocelluloses based fermentation process. *Ralstonia eutropha*, one of the most powerful and natural producers of polyhydroxyalkanoates (PHAs), has extensively been examined for the production of bio-based chemicals, fuels, and polymers. However, to the best of our knowledge, lignocellulosic feedstock has not been employed for *R. eutropha* probably due to its narrow spectrum of substrate utilization. Thus, *R. eutropha* engineered to utilize xylose should be useful in the development of microbial process for bio-based products from lignocellulosic feedstock.

**Results:**

Recombinant *R. eutropha* NCIMB11599 expressing the *E. coli xylAB* genes encoding xylose isomerase and xylulokinase respectively, was constructed and examined for the synthesis of poly(3-hydroxybutyrate) [P(3HB)] using xylose as a sole carbon source. It could produce 2.31 g/L of P(3HB) with a P(3HB) content of 30.95 wt% when it was cultured in a nitrogen limited chemically defined medium containing 20.18 g/L of xylose in a batch fermentation. Also, recombinant *R. eutropha* NCIMB11599 expressing the *E. coli xylAB* genes produced 5.71 g/L of P(3HB) with a P(3HB) content of 78.11 wt% from a mixture of 10.05 g/L of glucose and 10.91 g/L of xylose in the same culture condition. The P(3HB) concentration and content could be increased to 8.79 g/L and 88.69 wt%, respectively, when it was cultured in the medium containing 16.74 g/L of glucose and 6.15 g/L of xylose. Further examination of recombinant *R. eutropha* NCIMB11599 expressing the *E. coli xylAB* genes by fed-batch fermentation resulted in the production of 33.70 g/L of P(3HB) in 108 h with a P(3HB) content of 79.02 wt%. The concentration of xylose could be maintained as high as 6 g/L, which is similar to the initial concentration of xylose during the fed-batch fermentation suggesting that xylose consumption is not inhibited during fermentation. Finally, recombinant *R. eutorpha* NCIMB11599 expressing the *E. coli xylAB* gene was examined for the production of P(3HB) from the hydrolysate solution of sunflower stalk. The hydrolysate solution of sunflower stalk was prepared as a model lignocellulosic biomass, which contains 78.8 g/L of glucose, 26.9 g/L of xylose, and small amount of 4.8 g/L of galactose and mannose. When recombinant *R. eutropha* NCIMB11599 expressing the *E. coli xylAB* genes was cultured in a nitrogen limited chemically defined medium containing 23.1 g/L of hydrolysate solution of sunflower stalk, which corresponds to 16.8 g/L of glucose and 5.9 g/L of xylose, it completely consumed glucose and xylose in the sunflower stalk based medium resulting in the production of 7.86 g/L of P(3HB) with a P(3HB) content of 72.53 wt%.

**Conclusions:**

*Ralstonia eutropha* was successfully engineered to utilize xylose as a sole carbon source as well as to co-utilize it in the presence of glucose for the synthesis of P(3HB). In addition, *R. eutropha* engineered to utilized xylose could synthesize P(3HB) from the sunflower stalk hydrolysate solution containing glucose and xylose as major sugars, which suggests that xylose utilizing *R. eutropha* developed in this study should be useful for development of lignocellulose based microbial processes.

## Background

In recent years, the development of biomass-derived chemicals and polymers has been facilitated with increasing concerns on fossil fuel’s depletion and environmental problems such as global warming [1]. Biomass-based processes for the production of valuable chemicals and polymers are considered as carbon neutral processes that emit less carbon dioxide than petroleum-based processes if biomasses are efficiently converted into biosugars for microbial fermentation and into chemicals through direct chemical process [2]. However, lowering high production cost of biomass-derived chemicals and polymers still remains as prerequisites for commercialization of these products. Since feedstock costs account for more than 30 % of the total cost of bio-based products, many studies have focused on searching inexpensive and appropriate feedstock to reduce the entire production cost of bio-based products from renewable biomass resources [3, 4].

Corn and sugarcane have intensively been utilized for the production of biomass based chemicals and polymers since they are most efficiently converted into biosugars for microbial fermentation. However, use of these edible resources in biochemical processes has raised ethical issues. Unlike them, lignocellulosic biomasses surrounded by lignin that provides a robust wall to the hemicellulose-cellulose framework, are the most abundant resources on earth without competing with human food supply [5].

Lignocellulosic biomass mainly consists of cellulose, hemicelluloses, and lignin [4]. Since xylose is the main component of hemicelluloses, a lot of studies have been conducted to utilize this monosaccharide as fermentation feedstock to produce valuable biochemical products such as ethanol [6], 3-hydroxypropionic acid [7], 1,3-propanediol [8], 2,3-butanediol [9], and cadaverine [10]. One of the main barriers in utilizing xylose is that many industrially important microbial strains cannot utilize xylose as a sole carbon source and cannot efficiently utilize xylose when glucose, the most abundant sugar in cellulose in lignocellulosic biomass, is present along with xylose due to strong catabolic repression [11]. The *Escherichia colixylA* and *xylB* genes, encoding xylose isomerase and xylulokinase respectively, has been the most frequently employed genes to construct the xylose utilization pathway in microorganisms for the enhanced utilization of xylose as a carbon source [12–14].

Sunflower is the fourth oil-seeds source covering worldwide more than 26 million hectares cultivated land surface, which has been estimated to 3–7 tons of dry biomass per hectare [15]. After seed harvesting, stalk are left as wastes in the fields and usually eliminated by burning, which might cause additional environmental problems. Only a few sunflower stalk are being utilized as raw materials for paper pulp production [16] and bioethanol production [17]. Since sunflower stalk contain fermentable sugars such as glucose and xylose as main sugars along with rather small amount of galactose, mannose, and arabinose researches have been conducted to develop appropriate pretreatment and hydrolysis methods to utilize sunflower stalk as fermentation feedstock [18]. For example, 35.8 g of glucose, 19.7 g of hemicellulosic sugars have been produced as fermentable sugars by 100 g treatment [18]. One of the promising methods for pretreatment of sunflower stalk involves the hydrothermal pretreatment process in which C5 sugars are released from lignocellulosic biomass at relatively low temperature, usually ranging from 170 to 180 °C, without further degradation. The following enzymatic degradation of cellulose to glucose at higher temperature (190–200 °C) would result in a high sugar yield [19]. Furthermore, a more simplified hydrothermal process was examined for sunflower stalk pretreatment [18], in which, high glucose yields ranging from 67.0 to 90.0 % could be obtained by one-step hydrothermal pretreatment of sunflower stalk at 180–200 °C [18].

Polyhydroxyalkanoates (PHAs) are naturally synthesized biopolyesters that are accumulated in many bacteria as carbon and reducing power storage material [20, 21]. All the monomers of PHAs, more than 150 kinds of which have been identified up to date, are generated through inherent intermediates of metabolic pathways of host strains from the structurally related or unrelated carbon sources [20, 21].

The material properties of PHAs are highly dependent on the types and compositions of their monomer components, which generally are 3-, 4-, 5- or 6-hydroxycarboxylic acids in natural PHA producing bacteria. Recently, monomer spectrum of PHAs has also successfully been expanded to include 2-hydroxyacids such as lactate and 2-hydroxybutyrate as PHA monomers by metabolically engineered bacteria expressing engineered PHA synthase [22–25].

Among the various members of PHAs, poly(3-hydroxybutyrate) [P(3HB)] is one of the best characterized PHA for its efficient production in natural and recombinant bacteria such as *Ralstonia eutropha* and recombinant *Escherichia coli* along with characterization of its material properties for possible application as a general performance polymer. As a results, the cost-effective production of P(3HB) has been much examined to commercialize P(3HB) as a promising biomass-driven polymer material to substitute chemically synthesized polymers [20, 21, 25].

*Ralstonia eutropha* (recently renamed *Cupriavidus necator*) has been used as a host strain for the production of PHAs such as P(3HB), poly(3-hydroxybutyrate-co-3-hydroxyvalerate)[P(3HB-co-3HV)], and other novel PHAs since it is one of the microorganisms that most efficiently synthesize PHAs consisting of various monomers that can be derived from its inherent and engineered metabolic pathways [20, 21, 25].

Thus, in this study, *R. eutropha* NCIMB11599 strain that can efficiently utilize glucose as a carbon source has been further engineered to use xylose as a carbon source by the introduction of xylose utilization pathway of *E. coli* for the production of P(3HB) from sunflower hydrolysate solution containing glucose and xylose as major sugars. Recombinant *R. eutropha* NCIMB11599 expressing *E. colixylAB* genes has been examined for the production of P(3HB) in the culture media containing xylose as a sole carbon source and a mixture of glucose and xylose. Furthermore, sunflower stalk hydrolysates was examined as a biomass-derived bio-sugars for the production of P(3HB) in *R. eutropha* NCIMB11599 expressing *E. colixylAB* genes.

## Results and discussions

### Construction of recombinant *R. eutropha* able to utilize xylose as a carbon source

**Fig. 1:**
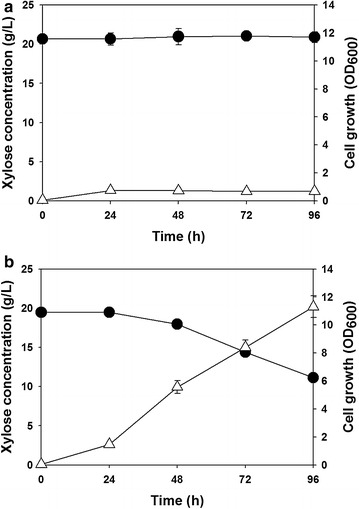
Time profiles of flask-cultures of **a** wild-type *R. eutropha* NCIMB11599, **b** Recombinant *R. eutropha* (pKM212-XylAB) in sole carbon of xylose based medium for the synthesis of P(3HB). (Symbols are: *filled circle*, xylose concentration; *open triangle*-*up*, cell growth or cell dry weight; *open square*, P(3HB) concentration; *open diamond*, P(3HB) content)

*Ralstonia eutropha* NCIMB11599 was firstly examined for the production of P(3HB) using xylose as a sole carbon source. As shown in Fig. 1a, *R. eutropha* NCIMB11599 could not grow using xylose as a sole carbon source when it was cultured in a chemically defined MR medium containing 20.39 g/L xylose as previously reported by the genome analysis of *R. eutropha* suggesting its narrow range of substrate usage that cannot support xylose utilization [26–29].

**Fig. 2:**
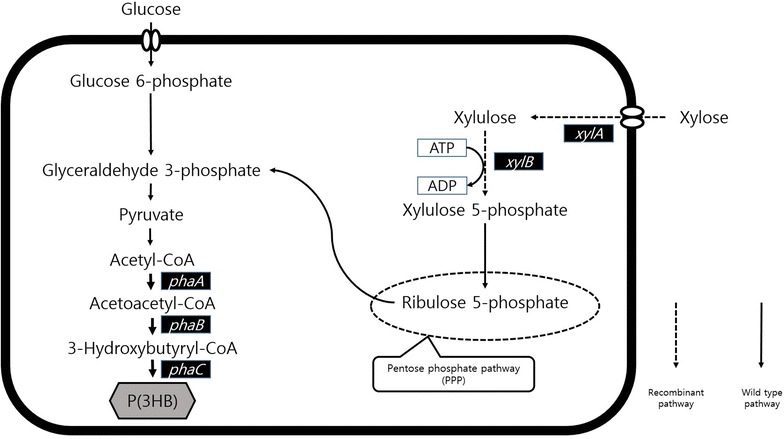
Metabolic pathways for biosynthesis of P(3HB) in recombinant *R. eutropha* strain from glucose and xylose as carbon sources used in this study. The overall metabolic pathway is shown together with the introduced metabolic pathways for the production of P(3HB). Xylulose 5-phosphate is generated by *E. coli* xylose isomerase (xylA) and xylulokinase (xylB). 3-Hydroxybutyryl-CoA is generated by *R. eutropha* β-ketothiolase (phaA) and acetoacetyl-CoA reductase (phaB). In *R. eutropha* strain, all the genes involved in PHA biosynthesis are in the chromosomal DNA except the *xylAB* gene encoding *E. coli* xylose isomerase and xylulokinase, which is additionally expressed by the introduction of pKM212-XylAB

Recombinant *R. eutropha* NCIMB11599 (pKM212-XylAB) expressing the *E. coli xylAB* genes was constructed to make this strain utilize xylose as a sole carbon source. The metabolic pathway of recombinant *R. eutropha* NCIMB11599 (pKM212-XylAB) is illustrated in Fig. 2. Recombinant *R. eutropha* NCIMB11599 (pKM212-XylAB) cultured in chemically defined MR medium containing 19.25 g/L of xylose, could consistently consumed xylose during the flask culture and grew up to an OD_600_ of 11 (Fig. 1b). In addition, when it was cultured in nitrogen free chemically defined MR-N medium containing 19.25 g/L of xylose, it accumulated P(3HB) to a concentration of 0.45 g/L with a P(3HB) content of 72.58 wt% and yield of 30.31 wt% (Table 1).

**Table 1:** Flask culture of *R. eutropha* NCIMB11599 expressing *E. coli xylAB* genes in nitrogen free chemically defined MR-N medium containing xylose as a sole carbon source

Strain	Carbon source	DCW (g/L)	P(3HB) concentration (g/L)	P(3HB) content (wt%)
*R. eutropha* pKM212-XylAB	Xylose	0.64 ± 0.01	0.45 ± 0.04	72.58 ± 2.80

### Examination of a mixture of glucose and xylose as carbon sources in recombinant *R. eutropha* NCIMB11599 (pKM212-XylAB)

Recombinant *R. eutropha* NCIMB11599 (pKM212-XylAB) was examined for its growth characteristics in MR medium containing both glucose and xylose as carbon sources. Although several microbial strains such as *Lactobacillus brevis*, and *Lactobacillus plantarum*, which are not able to utilize xylose as a sole carbon source, have been reported to be able to utilize xylose in the presence of glucose [30], it was found out that *R. eutropha* NCIMB11599 could not still consume xylose even in the presence of glucose. When *R. eutropha* NCIMB11599 was cultured in MR medium containing 9.78 g/L of glucose and 10.43 g/L of xylose, it completely consumed glucose in 72 h to grow up to an OD_600_ of 21.12 (data not shown). However, the concentration of xylose was maintained as high as the initial concentration throughout the culture.

**Fig. 3:**
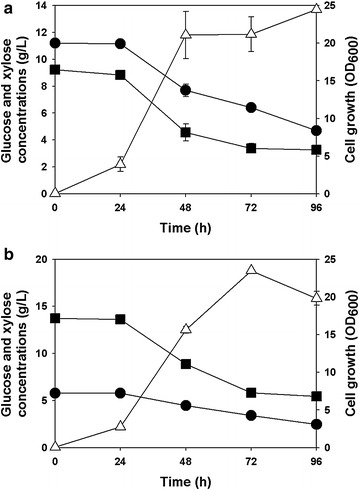
Time profiles of flask cultures of recombinant *R. eutropha* NCIMB11599 (pKM212-XylAB) in MR medium containing mixed sugars of glucose and xylose as carbon sources. **a** 9.25 g/L of glucose and 10.97 g/L of xylose were used as carbon sources **b** 13.76 g/L of glucose and 5.78 g/L of xylose were used as carbon sources. (Symbols are: *filled square*, glucose concentration; *filled circle*, xylose concentration; *open triangle*-*up*, cell growth)

On the other hand, recombinant *R. eutropha* NCIMB11599 (pKM212-XylAB) could utilize both glucose and xylose when it was cultured in MR medium containing these two substrates. As shown in Fig. 3a, *R. eutropha* NCIMB11599 (pKM212-XylAB) consumed 5.97 g/L of glucose and 6.51 g/L of xylose, growing up to an OD_600_ of 21.14, from 9.16 g/L of glucose and 11.43 g/L of xylose, respectively. The rates of glucose and xylose utilization were 0.0622 g/L/h and 0.0678 g/L/h, respectively. By the way, when recombinant *R. eutropha* NCIMB11599 (pKM212-XylAB) was cultured in the medium initially containing 13.68 g/L of glucose and 5.76 g/L of xylose, it consumed 8.29 g/L of glucose and 3.32 g/L of xylose, with the rates of glucose and xylose utilization of 0.0864 g/L/h and 0.0346 g/L/h, respectively, to grow up to an OD_600_ of 19.01 (Fig. 3b). The rate of xylose utilization by recombinant *R. eutropha* NCIMB11599 (pKM212-XylAB) tended to decrease as the initial ratio of glucose to xylose in the medium increased. In contrast, the increased substrate ratio could speed up the glucose consumption rate of the host strain.

When recombinant *R. eutropha* NCIMB11599 (pKM212-XylAB) was cultured in a nitrogen free chemically defined MR-N medium containing both glucose and xylose as carbon sources, it exhibited higher P(3HB) production capacities than that obtained by the flask culture using xylose as a sole carbon source. In addition, production of P(3HB) was enhanced as the initial ratio of glucose to xylose in MR-N medium increased. The concentration of P(3HB) and P(3HB) content obtained by recombinant *R. eutropha* NCIMB11599 (pKM212-XylAB) cultured in MR-N medium containing 9.85 g/L of glucose and 10.01 g/L of xylose were 1.49 g/L and 86.58 wt%, respectively. The consumed glucose and xylose were 2.21 g/L and 3.27 g/L of xylose, respectively. On the other hand, 1.65 g/L of P(3HB) and 93.33 wt% of P(3HB) content were obtained by recombinant *R. eutropha* NCIMB11599 (pKM212-XylAB) cultured in MR-N medium containing 14.36 g/L of glucose and 5.63 g/L of xylose by consuming 2.23 g/L of glucose and 1.09 g/L of xylose (Table 2). Also, the P(3HB) yields of 49.96 wt% obtained in MR-N medium containing 14.36 g/L of glucose and 5.63 g/L of xylose was higher than that of 23.54 wt% obtained in MR-N medium containing 9.85 g/L of glucose and 10.01 g/L of xylose. Taken together, it seems to be that *R. eutropha* NCIMB11599 (pKM212-XylAB) still favors glucose to xylose in terms of substrate utilization and P(3HB) production capacity even though it has the functional xylose-utilizing pathway.

**Table 2:** Results of analysis of bacterial growth, P(3HB) concentration, and P(3HB) contents by recombinant *R. eutropha* NCIMB11599 (pKM212-XylAB) using mixed carbon sources during flask cultivations using MR-N medium containing glucose and xylose as carbon sources

Strain	Glucose (g/L)	Xylose (g/L)	DCW (g/L)	P(3HB) concentration (g/L)	P(3HB) content (wt%)
*R. eutropha* (pKM212-XylAB)	9.25	10.97	1.49 ± 0.15	1.29 ± 0.10	86.58 ± 0.52
	13.76	5.78	1.65 ± 0.03	1.54 ± 0.11	93.33 ± 0.65

The mechanism of xylose uptake by *R. eutropha* has not yet been clearly understood. The sequence similarity search by the protein BLAST indicates that *R. eutropha* seems not to possess a possible xylose transporter like *E. coli*. There are several reports suggesting bacterial xylose uptake through different mechanisms. A facilitated diffusion mechanism has been reported to be functional in Lactobacillus pentosus for xylose transportation [31]. On the other hand, *Corynebacterium glutamicum* has been suggested to possess several different mechanisms for xylose transportation [32]. Taken together, further studies are required in order to understand the xylose uptake mechanisms in *R. eutropha*.

### Examination of P(3HB) production in recombinant *R. eutropha* NCIMB11599 (pKM212-XylAB) from a mixture of glucose and xylose as carbon sources in fermentation

**Fig. 4:**
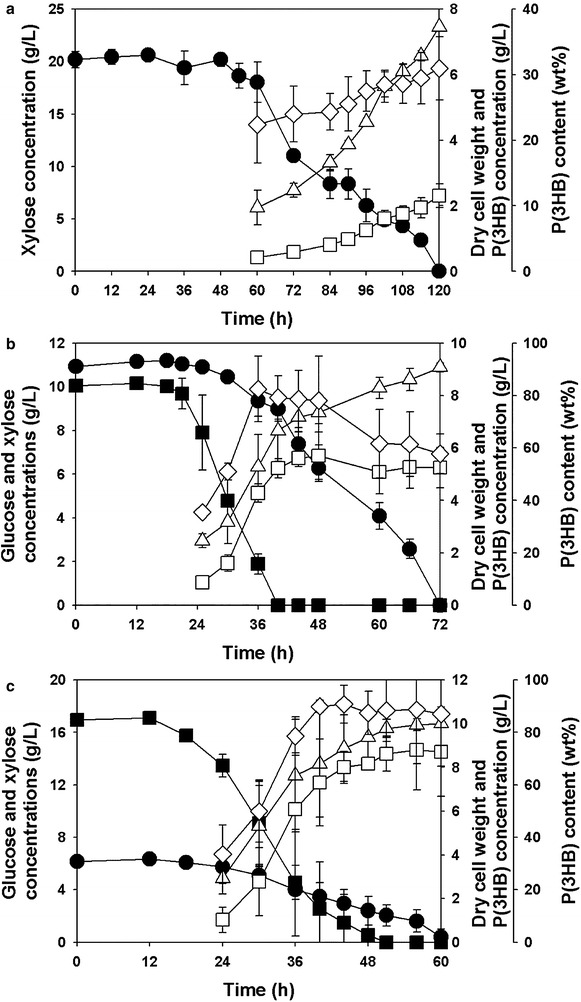
Time profiles of batch fermentations of **a** recombinant *R. eutropha* NCIMB11599 (pKM212-XylAB) in MR-B medium containing 20.18 g/L of xylose as a sole carbon source, **b** recombinant *R. eutropha* NCIMB11599 (pKM212-XylAB) in MR-B medium containing 10.05 g/L of glucose and 10.91 g/L of xylose as carbon sources and **c** recombinant *R. eutropha* NCIMB11599 (pKM212-XylAB) in MR-B medium containing 16.94 g/L of glucose and 6.15 g/L of xylose as carbon sources. (Symbols are: *filled square*, glucose concentration; *filled circle*, xylose concentration; *open triangle*-*up*, dry cell weight; *open square*, P(3HB) concentration; *open diamond*, P(3HB) content)

The growth and P(3HB) production profiles of recombinant *R. eutropha* NCIMB11599 (pKM212-XylAB) was further examined in batch fermentations in a 2.5 L jar fermentor (CNS Co. Ltd., Korea) using xylose or mixtures of xylose and glucose as carbon sources, respectively. When recombinant *R. eutropha* NCIMB11599 (pKM212-XylAB) was cultured in MR-B medium containing 20.18 g/L of xylose, it completely consumed the substrate in 120 h (Fig. 4a). Considering that recombinant *R. eutropha* NCIMB11599 (pKM212-XylAB) was able to utilize glucose within 12 h when it was cultured in MR-B medium containing 20 g/L of glucose as a sole carbon source (data not shown), it showed relatively a long lag phase of 48 h in the xylose fermentation. In addition, it took three times longer to consume 20.18 g/L of xylose than to consume the same amount of glucose (data not shown). After the lag phase, *R. eutropha* NCIMB11599 (pKM212-XylAB) gradually consumed xylose and then grew up to a DCW (dry cell weight) of 7.46 g/L in 120 h (Fig. 4a). The highest P(3HB) concentration P(3HB) content, and P(3HB) yield achieved in 120 h were 2.31 g/L, 30.95 wt%, and 11 wt% (g polymer/g xylose), respectively. The xylose consumption rate was 0.164 g/L/h.

When 10.91 g/L of xylose and 10.05 g/L of glucose were used as carbon sources, recombinant *R. eutropha* NCIMB11599 (pKM212-XylAB) completely consumed glucose in 40 h and xylose in 72 h, respectively, resulting in the highest DCW of 9.08 g/L in 72 h (Fig. 4b). While *R. eutropha* NCIMB11599 (pKM212-XylAB) was able to utilize glucose in 25 h, it began to utilize xylose in 30 h after inoculation. The highest concentration of P(3HB), P(3HB) content, P(3HB) yield, P(3HB) productivity of 5.71 g/L, 78.11 wt%, 40.0 wt% (g polymer/g glucose + xylose), and 0.12 g/L/h, respectively, were obtained by consuming 10.91 g/L of glucose and 4.19 g/L of xylose in 48 h. The rates of glucose and xylose consumption were 0.25 g/L/h and 0.15 g/L/h, respectively. Although the mixed-substrates fermentation was completed within a much shorter time than the xylose fermentation, the rate of xylose consumption decreased in the presence of glucose.

In addition, fermentation of recombinant *R. eutropha* NCIMB11599 (pKM212-XylAB) in MR-B medium containing 6.15 g/L of xylose and 16.94 g/L of glucose resulted in different profiles of cell growth, substrate consumption rates, and P(3HB) production from the previous fermentations (Fig. 4a, b, and c). Recombinant *R. eutropha* NCIMB11599 (pKM212-XylAB) began to utilize glucose in 12 h after inoculation and then completely consumed the substrate in 51 h. On the other hand, it was able to consume xylose in 24 h after inoculation and then completely consumed the substrate in 60 h, growing to the highest DCW of 10.01 g/L. The highest concentration of P(3HB) of 8.79 g/L was obtained in 56 h by consuming 16.94 g/L of glucose and 3.97 g/L of xylose with in a P(3HB) content of 88.69 wt%, a P(3HB) yield of 42.0 wt%, and a productivity of 0.15 g/L/h, respectively. The rates of glucose and xylose consumption were 0.33 g/L/h and 0.09 g/L/h, respectively. While the consumption rate of glucose increased by 31 % than that obtained with 10.05 g/L of glucose and 10.91 g/L xylose, the consumption rate of xylose decreased by 40 %.

**Fig. 5:**
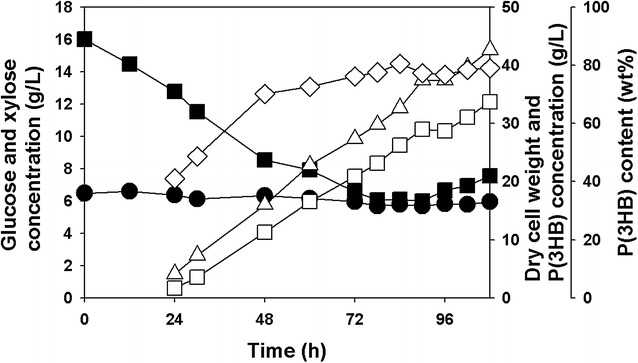
Time profiles of fed-batch fermentations of recombinant *R. eutropha* NCIMB11599 (pKM212-XylAB) in MR-B medium containing 16.01 g/L of glucose and 7.48 g/L of xylose as carbon sources. (Symbols are: *filled square*, glucose concentration; *filled circle*, xylose concentration; *open triangle*-*up*, dry cell weight; *open square*, P(3HB) concentration; *open diamond*, P(3HB) content)

The growth and P(3HB) production profiles of recombinant *R. eutropha* NCIMB11599 (pKM212-XylAB) were further investigated in fed-batch fermentations in a 2.5 L jar fermentor (Fig. 5). The initial concentrations of glucose and xylose were 16.01 and 7.48 g/L, respectively. While the DCW and P(3HB) concentration gradually increased, the P(3HB) content reached to the highest point (80.51 wt%) at 84 h and was not significantly changed during fermentation. The P(3HB) yield for the batch phase and that for the feeding phase were 22.63 and 35.89 wt%, respectively, resulting in the overall yield of 34.87 wt%. Considering that the ratio of xylose to glucose in the feeding solution was 1:3 and the consumption rate of xylose (0.09 g/L/h) was less than one-third of that of glucose (0.33 g/L/h) in the previous batch fermentation, it was interesting that the xylose concentration was maintained at about 6 g/L, instead of being accumulated during the fed-batch fermentation. It is interesting to note that this result was mainly due to the increased consumption rate of xylose during the feeding phase (0.27 g/L/h).

Since the highest cell growth, P(3HB) concentration, content, weight yield, and productivity achieved by batch fermentations of recombinant *R. eutropha* NCIMB11599 (pKM212-XylAB) increased as the initial ratio of glucose to xylose in the culture medium increased, it could be concluded that glucose is still a preferred substrate to xylose for its cell growth and P(3HB) production, even though the engineered *R. eutropha* NCIMB11599 (pKM212-XylAB) successfully utilized xylose as a sole carbon source as well as co-utilized it in the presence of glucose. This phenomenon is quite common in microbial species including *E. coli*, *Saccharomyces cerevisiae*, *Bacillus subtilis*, *Pseudomonas putida*, *Lactococcus lactis*, *Lactobacillus casei*, and *C. glutamicum* [33–36]. Among them, *C. glutamicum*, which is not a native xylose utilizing organism, has also been investigated for xylose utilization by introducing the *E. coli* xylAB genes used in this study [33]. It was reported that expression of the *xylAB* genes is enough for the construction of recombinant *C. glutamicum* to consume xylose as a carbon source without further engineering to improve xylose consumption such as introduction of heterologous transporters for xylose and overexpression of native transporters. However, engineered strains of *C. glutamicum* to utilize xylose still preferred glucose when it was cultured in a medium containing both sugars, glucose and xylose. Recombinant *C. glutamicum* that has been engineered to efficiently use xylose as a carbon source still showed twice higher consumption rate of glucose than that of xylose when it was cultured in a medium containing 20 g/L of glucose and 10 g/L of xylose [33].

On the other hand, previous studies have focused on using native xylose-utilizing hosts such as *E. coli* [37] and *Burkholderia cepacia* [38] for the production of PHAs from xylose. We successfully developed an engineered *R. eutropha* to utilize xylose as a sole carbon source as well as co-utilize xylose in the presence of glucose. However, it was observed that, like other well-known microbial hosts mentioned above, recombinant *R. eutropha* engineered in this study is still carbon catabolite repression (CCR)-sensitive in the presence of glucose. To overcome this problem, it would be necessary to apply the engineering approaches examined in other strains into *R. eutropha* for improving xylose catabolism along with the reduction of CCR [36, 39–41]. For example, enhanced expression of the transketolase operon in the pentose phosphate pathway by replacing its native promoter with the strong sod promoter in *C. glutamicum* could lead to the increase of the product yield from xylose [42]. In addition, an engineered *S. cerevisiae* overexpressing the transaldolase gene exhibited a higher xylose consumption rate than its parental strain when it was cultured in the medium containing both glucose and xylose [43]. Introduction of heterologous xylose transporters may also be a good strategy to increase xylose consumption rate along with reduced CCR [33, 34].

In addition, production of different types of PHA copolymers from xylose can be achieved by employing PHA synthase with a promiscuous substrate specificity and by introducing additional metabolic pathways to supply various hydroxyacyl-CoA (HA-CoA) with different carbon numbers and R-pending groups to PHA synthase [44]. Since xylulose 5-phosphate, synthesized from xylose by the enzymatic reaction of XylA and XylB, may enter into the pentose phosphate pathway yielding glyceraldehyde 3-phosphate, which is an intermediate of glycolysis, most of engineering strategies for PHA copolymers based on glycolysis would be applicable for the production of PHA copolymers from xylose as a carbon source.

### Biosynthesis of P(3HB) in recombinant *R. eutropha* NCIMB11599 (pKM212-XylAB) from sunflower stalk hydrolysate solution

To examine the possible application of recombinant *R. eutropha* NCIMB11599 (pKM212-XylAB) in a lignocellulose-based PHA production process, sunflower stalk has been chosen as a raw material for biosugar. The hydrolysate solution of sunflower stalk was prepared by hydrothermal pretreatment and subsequent hydrolysis of the raw material. The sunflower stalk hydrolysate solution contained 8.7 wt% glucose, 2.4 wt% hemicellulosic sugars, 0.006 wt% furfural, and ash. Hemicellulosic sugars contained 85.1 wt% of xylose, 0.2 wt% of galactose, and 14.7 wt% of mannose, respectively. The hydrolysate solution of sunflower stalk may be successfully employed for microbial fermentation process since it contains quite low concentrations of possible inhibitors such as acetic acid, furfural, and 5-hydroxy-methyl-2-furaldehyde (HMF), all of which are known to be generated from hydrothermal pretreatment of lignicellulosic biomass.

**Fig. 6:**
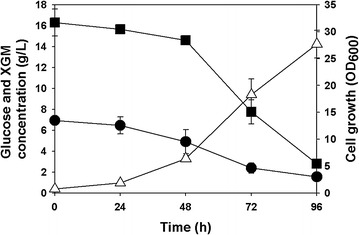
Time profiles of flask cultures of recombinant *R. eutropha* NCIMB11599 (pKM212-XylAB) in MR medium containing sunflower stalk hydrolysate solution as a carbon source. The initial XGM is composed of 85.13 % xylose, 0.23 % galactose, and 14.64 % mannose. (Symbols are: *filled square*, glucose concentration; *filled circle*, xylose, galactose, and mannose (XGM) concentration; *open triangle*-*up*, cell growth)

Growth of recombinant *R. eutropha* NCIMB11599 (pKM212-XylAB) from sunflower stalk hydrolysate solution was firstly examined in flask cultures, in which MR medium containing 1:5 diluted hydrolysate solution of sunflower stalk was used. Recombinant *R. eutropha* NCIMB11599 (pKM212-XylAB) could grow to an OD_600_ of 27.67 by consuming 13.49 g/L of glucose and 4.37 g/L of xylose in 96 h (Fig. 6). Also, galactose and mannose existing in sunflower stalk hydrolysate solution was completely consumed by recombinant *R. eutropha* NCIMB11599 (pKM212-XylAB) in 96 h as previous reported [45, 46]. When this strain was cultured in MR-N medium containing 1:5 diluted hydrolysate solution of sunflower stalk, it produced 2.42 g/L of P(3HB) with a P(3HB) content of 75.42 wt% by consuming 5.61 g/L of glucose, 0.431 g/L of xylose, 0.00101 g/L of galactose, and 0.0744 g/L of mannose. The P(3HB) yield was 39.50 wt% (g polymer/g glucose + xylose + galactose + mannose) which is higher than that synthesized by recombinant *R. eutropha* NCIMB11599 (pKM212-XylAB) in MR-N medium containing 10.05 g/L of glucose and 10.91 g/L of xylose, but lower than that in MR-N medium containing containing 16.94 g/L of glucose and 6.15 g/L of xylose (Table 3).

**Table 3:** Results of analysis of bacterial growth, P(3HB) concentration, and P(3HB) contents by recombinant *R. eutropha* NCIMB11599 (pKM212-XylAB) during flask cultivation in MR-N medium containing sunflower stalk hydrolysate solution as a carbon source

Strain	Carbon source	DCW (g/L)	P(3HB) concentration (g/L)	P(3HB) content (wt%)
*R. eutropha* (pKM212-XylAB)	Sunflower stalk^a^	2.46 ± 0.04	2.09 ± 0.01	84.96 ± 0.89

^a^Initial concentrations of carbon sources in the medium were 15.55 g/L of glucose, 5.41 g/L of xylose, 0.01 g/L of galactose, and 0.92 g/L of mannose

**Fig. 7:**
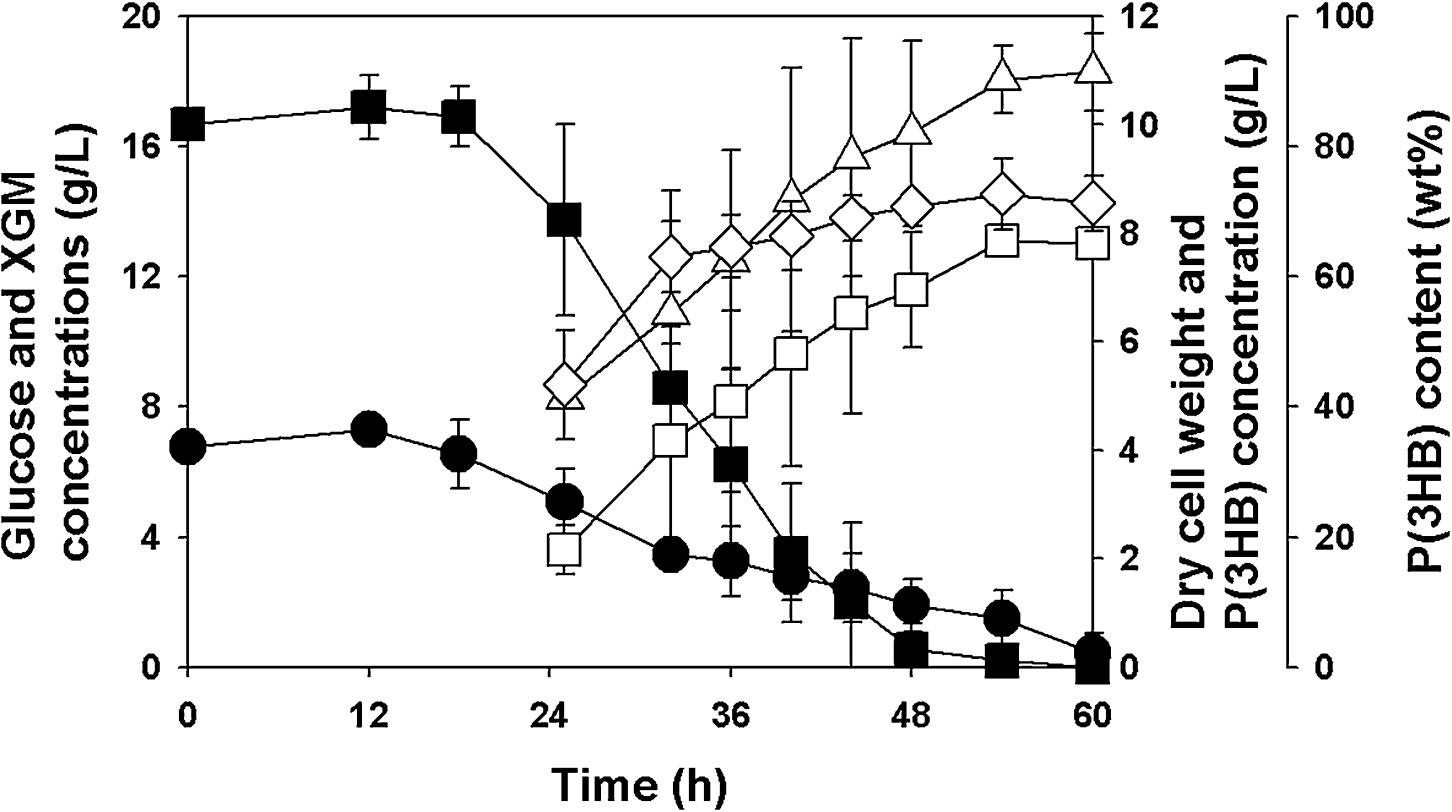
Time profiles of batch fermentation of recombinant *R. eutropha* NCIMB11599 (pKM212-XylAB) in MR-B medium containing sunflower stalk hydrolysate. The initial XGM is composed of 85.13 % xylose, 0.23 % galactose, and 14.64 % mannose. (Symbols are: *filled square*, *glucose concentration; filled circle*, xylose, galactose, and mannose (XGM) concentration; *open triangle*-*up*, dry cell weight; *open square*, P(3HB) concentration; *open diamond*, P(3HB) content)

Batch fermentation of recombinant *R. eutropha* NCIMB11599 (pKM212-XylAB) was carried out in a 2.5 L jar fermentor with MR-B medium using the sunflower stalk hydrolysate solution as a carbon source. The time profiles of cell growth and P(3HB) production are shown in Fig. 7. Recombinant *R. eutropha* NCIMB11599 (pKM212-XylAB) grew to a DCW of 10.97 g/L and completely consumed all fermentable sugars in 60 h. The highest P(3HB) concentration of 7.86 g/L was achieved with a P(3HB) content of 72.53 wt% and a P(3HB) yield of 34.52 wt% in 54 h.

As the initial composition of fermentable sugars in the sunflower stalk based medium was similar to that in the mixed sugar based medium (about 16.94 g/L of glucose and 6.15 g/L of xylose), recombinant *R. eutropha* showed similar profiles of cell growth and substrate consumption in both media. However, the highest concentration of P(3HB) synthesized by recombinant *R. eutropha* (pKM212-XylAB) in the sunflower stalk based medium was lower than that in the mixed sugar based medium even though it grew slightly better on the former medium than on the latter medium in terms of the highest DCW. In other words, the sunflower stalk hydrolysate solution can be a better fermentation feedstock for *R. eutropha* (pKM212-XylAB) than the mixture of glucose and xylose in terms of cell growth, but not of P(3HB) production. As previously reported by the studies on the examination of lignocellulosic biomass as raw materials for fermentation, unknown components present in the hydrolysate solution seemed to negatively affect the host cell to synthesize target products [17, 47, 48]. *R. eutropha* has also been examined for PHA production using lignocellulosic raw materials [49]. In this study, the alkali pretreated hydrolysates of rice paddy straw, sunflower husk, soybean straw, and wood straw have been examined for the cell growth and P(3HB) production by *R. eutropha*, in which the hydrolysate of rice paddy straw resulted in the highest P(3HB) concentration, yield, and weight. It was found out that both the cell growth and P(3HB) synthesis were hindered by the paddy straw hydrolysate, whereas sunflower hydrolysate solution used in present study did not significantly affect cell growth and P(3HB) synthesis efficiency. This might be from the different characteristics of sunflower stock and hydrothermal pretreatment process employed in this study.

Since only a few lignocelluloses have been examined for PHA production in recombinant and natural *R. eutropha* strains, it is difficult to evaluate economic feasibility of PHA production process due to the limited data on the effect of lignocellulosic feedstock cost on the entire production cost. Thus, investigation of more raw materials from different lignocelluloses for PHA production are needed to evaluate their economic feasibility, from which fermentable sugars are obtained by employing appropriate pretreatment and hydrolysis methods. In addition, availability of raw materials in the area of production process should be considered because transportation cost may exceed production cost if a process is constructed far away from the farmland along with the digestibility and efficiency of lignocelluloses in fermentation process after hydrolysis process.

Finally, all of process parameters including efficiency of microbial producers, fermentation types, purification techniques, process design, capital costs, and operation costs should be taken into consideration for the development of economically feasible process for the production of PHA based on lignocellulosic materials.

## Conclusions

*Ralstonia eutropha* was successfully engineered to utilize xylose, the second abundant sugar from lignocellulosic biomass, by the introduction of a heterologous xylose-utilizing pathway from *E. coli*. While the wild-type *R. eutropha* could not utilize xylose as a sole carbon source and it in the presence of glucose, recombinant *R. eutropha* expressing the *E. coli xylAB* genes could successfully utilize xylose as a sole carbon source resulting in the production of P(3HB). In addition, this strain could co-utilize xylose even in the presence of glucose. Finally, it efficiently utilized the hydrolyzed glucose and xylose derived from sunflower stalk as a model lignocellulosic biomass resulting in the accumulation of P(3HB) with a high content. Further engineering of xylose utilizing pathway in *R. eutropha* should be useful for enhanced economic feasibility of lignocellulose based production of polymers, chemicals and fuels.

## Methods

### Bacterial strains, genes, and plasmids

*Escherichia coli* XL1-Blue (Stratagene Cloning Systems, La Jolla, CA, USA) was used for gene cloning. Plasmid pKM212-MCS, the expression vector containing the tac promoter has been described previously [50]. All DNA manipulations were performed following standard procedures [51]. The polymerase chain reaction (PCR) was performed using the C1000 Thermal Cycler (Bio-Rad, USA). Primers used in this study (5′-gaattcatgcaagcctattttgaccagc and 5′-ggtacc ttacgccattaatggcagaag) were synthesized at Bioneer (Daejeon, Korea). The pKM212-XylAB was constructed by cloning the *E. coli xylAB* genes amplified from *E. coli* XL1-Blue chromosomal DNA into pKM212-MCS at *Eco*RI and *Kpn*I sites. Construction of recombinant *R. eutropha* NCIMB11599 was carried out through mating with *E. coli* S17-1 harboring pKM212-XylAB, as previously described [52].

### Preparation of sunflower stalk hydrolysates

Hydrothermal treatment was carried out following the previous method with slight modifications [18]. A 700 g of powdered sunflower stalk and deionized water were added in a reactor vessel to reach a final weight of 8750 g, and left overnight. Hydrothermal treatment was performed at 190 °C for 5 min and was stirred at 800 rpm. The pretreated slurry was cooled down to room temperature by quickly dipping the reactor vessel in flowing tap water. The pretreated slurry and 4550 g of deionized water were mixed and transferred quantitatively to a cotton fabric bag, and centrifuged at 3727*g* for 1 h with a swing-type centrifuge to separate liquid fraction (LF) from solid fraction (SF). The SF was further treated to yield the sunflower stalk hydrolysates solution as bellows.

To initiate the hydrolysis of SF, 1600 g of deionized water was poured into a lab-scale fermenter (7 L) equipped with a helical type impeller, followed by the addition of 542.5 g of SF and 22.7 mL of Cellic CTec3 (150 FPU/ml). Enzymatic hydrolysis was performed in the fermenter at 50 °C and 200 rpm for 72 h. Sodium hydroxide solution (1 N) was added to adjust the pH at 5.5. The same amount of the substrate and enzyme was added at every 2 h for 5 times. Total amounts of SF and enzyme were 3256 g (equivalent to 1356 g dry raw biomass) and 136 ml, respectively. After 72 h of hydrolysis, the hydrolysates were separated by centrifugation, and the supernatant was recovered as a fermentable sugar. The monomeric sugars present in the fermentable sugar solution were determined using HPLC.

### Culture conditions

*Escherichia coli* XL1-Blue for general cloning studies was cultured at 37 °C in Luria–Bertani (LB) medium (containing per liter: 10 g tryptone, 5 g yeast extract and 5 g NaCl). *R. etropha* NCIMB11599 strain was cultured in a chemically defined MR medium containing 20.39 g/L of xylose at 30 °C in a rotary shaker at 200 rpm for 96 h and its recombinant strain *R. eutropha* NCIMB11599 (pKM212-XylAB) was cultured in the MR medium containing 19.25 g/L of xylose, a mixture of 11.42 g/L of xylose and 9.15 g/L of glucose, a mixture of 5.76 g/L of xylose and 13.68 g/L of glucose, or 1:5 diluted sunflower stalk hydrolysate solution as carbon source at 30 °C in a rotary shaker at 200 rpm for 96 h. The MR medium (pH 7.0) contains (per liter) 6.67 g KH_2_PO_4_, 4 g (NH_4_)_2_HPO_4_, 0.8 g MgSO_4_·7H_2_O, 0.8 g citric acid, and 5 ml trace metal solution. The trace metal solution contains (per liter of 0.5 M HCl) 10 g FeSO_4_·7H_2_O, 2 g CaCl_2_, 2.2 g ZnSO_4_·7H_2_O, 0.5 g MnSO_4_·4H_2_O, 1 g CuSO_4_·5H_2_O, 0.1 g (NH_4_)_6_Mo_7_O_24_·4H_2_O, and 0.02 g Na_2_B_4_O_7_·10H_2_O. A chemically defined nitrogen free MR medium (MR-N) was used for the production of P(3HB) by *R. eutropha* NCIMB11599 and recombinant *R. eutropha* NCIMB11599 (pKM212-XylAB). MR-N medium is the same as MR medium except that 4 g/L of (NH_4_)_2_HPO_4_ is replaced with 4 g/L of Na_2_HPO_4_. Kanamycin (Km, 300 μg/mL) was added to the culture medium for recombinant *R. eutropha* NCIMB11599 (pKM212-XylAB).

### Fermentations

Batch fermentations of recombinant *R. eutropha* (pKM212-XylAB) strain were carried out at 30 °C in 2.5 L jar fermentors (CNS Co. Ltd., Korea) containing 1 L of MR-B medium, the composition of which is the same as MR-N medium except that 0.5 g/L of (NH_4_)_2_HPO_4_ is supplemented. The initial amounts of carbon sources in the medium were 20.18 g/L of xylose, a mixture of 10.91 g/L of xylose and 10.05 g/L of glucose, a mixture of 6.15 g/L of xylose and 16.94 g/L of glucose, or 1:5 diluted sunflower stalk hydrolysate. Seed cultures (100 mL) were prepared in LB medium overnight at 30 °C. The culture pH was controlled at 6.8 by the automatic addition of 28 % (v/v) NH_4_OH and the agitation speed was controlled at 200 rpm. Cell growth was monitored by measuring OD_600_ using UV spectrometer.

Fed-batch fermentation of recombinant *R. eutropha* (pKM212-XylAB) strain was carried out at 30 °C in 2.5 L jar fermentors (CNS Co. Ltd., Korea) containing 1 L of MR-B medium supplemented with 7.48 g/L of xylose and 16.01 g/L of glucose. A seed culture (100 mL) was prepared in LB medium overnight at 30 °C. The feeding solution containing 375 g/L of glucose and 125 g/L of xylose was added to the medium to maintain the glucose concentration at from 6 to 8 g/L. The culture pH was controlled at 6.8 by the automatic addition of 6 N NaOH and the agitation speed was controlled at 200 rpm.

### Analytical methods

Concentrations of sugars and organic acids in the culture media were measured by high performance liquid chromatography (HPLC). The content and concentration of P(3HB) were determined by gas chromatography (GC) using Agilent 6890 N GC system (Agilent Technologies, CA, USA) equipped with Agilent 7683 automatic injector, flame ionization detector, and a fused silica capillary column (SPB™-5, 30 m × 0.32 mm ID, 0.25 μm film; Supelco, Bellefonte, PA, USA). About 30 mg of dried cell was subjected to methanolysis with benzoic acid as an internal standard in the presence of 15 wt% sulfuric acid. The resulting methyl ester of 3-hydroxybutyrate was analyzed by GC according to the method of previous report [53]. Cell concentration was defined as dry cell weight (DCW) per liter of culture broth. The P(3HB) content (wt%) was defined as the percentage of the ratio of P(3HB) concentration to cell concentration.

## Abbreviations

P(3HB): poly(3-hydroxybutyrate)
xylA: xylose isomerase
xylB: xylulokinase
CCR: carbon catabolite repression
